# Artificial Intelligence for COVID-19 Drug Discovery and Vaccine Development

**DOI:** 10.3389/frai.2020.00065

**Published:** 2020-08-18

**Authors:** Arash Keshavarzi Arshadi, Julia Webb, Milad Salem, Emmanuel Cruz, Stacie Calad-Thomson, Niloofar Ghadirian, Jennifer Collins, Elena Diez-Cecilia, Brendan Kelly, Hani Goodarzi, Jiann Shiun Yuan

**Affiliations:** ^1^Burnett School of Biomedical Sciences, University of Central Florida, Orlando, FL, United States; ^2^Department of Electrical and Computer Engineering, University of Central Florida, Orlando, FL, United States; ^3^A2A Pharmaceuticals, Cambridge, MA, United States; ^4^Atomwise Inc., San Francisco, CA, United States; ^5^Department of Chemistry and Biochemistry, University of Arizona, Tucson, AZ, United States; ^6^Department of Biochemistry and Biophysics, Helen Diller Family Comprehensive Cancer Center, University of California, San Francisco, San Francisco, CA, United States

**Keywords:** COVID-19, SARS-COV-2, drug, vaccine, artificial intelligence, deep learning

## Abstract

SARS-COV-2 has roused the scientific community with a call to action to combat the growing pandemic. At the time of this writing, there are as yet no novel antiviral agents or approved vaccines available for deployment as a frontline defense. Understanding the pathobiology of COVID-19 could aid scientists in their discovery of potent antivirals by elucidating unexplored viral pathways. One method for accomplishing this is the leveraging of computational methods to discover new candidate drugs and vaccines *in silico*. In the last decade, machine learning-based models, trained on specific biomolecules, have offered inexpensive and rapid implementation methods for the discovery of effective viral therapies. Given a target biomolecule, these models are capable of predicting inhibitor candidates in a structural-based manner. If enough data are presented to a model, it can aid the search for a drug or vaccine candidate by identifying patterns within the data. In this review, we focus on the recent advances of COVID-19 drug and vaccine development using artificial intelligence and the potential of intelligent training for the discovery of COVID-19 therapeutics. To facilitate applications of deep learning for SARS-COV-2, we highlight multiple molecular targets of COVID-19, inhibition of which may increase patient survival. Moreover, we present CoronaDB-AI, a dataset of compounds, peptides, and epitopes discovered either *in silico* or *in vitro* that can be potentially used for training models in order to extract COVID-19 treatment. The information and datasets provided in this review can be used to train deep learning-based models and accelerate the discovery of effective viral therapies.

## Introduction

Coronaviridae is a viral family responsible for causing pneumonia-like symptoms that has been a global threat since its first outbreak in 2002 (Jabeer Khan et al., 2020). Severe Acute Respiratory Disease (SARS) and Middle Eastern Respiratory Syndrome (MERS), emerging in 2002 and 2013, respectively, caused diseases marked by both gastrointestinal and pulmonary dysfunction (Hilgenfeld and Peiris, 2013). In 2019, SARS-COV-2 was the causative agent of a third Coronavirus outbreak and has been identified as the virus responsible for COVID-19, the symptoms of which range from those of the common cold to more severe respiratory failure (Kong W.-H. et al., 2020). Despite its having been declared a pandemic by the World Health Organization (WHO), COVID-19 has continued to spread and has infected at least 20 million individuals, reaching a death toll of over half a million at the time of this review (Worldometer, 2020).

While hospitals are resorting to trial and error tactics for COVID-19 drug discovery, Virtual Screening (VS) has emerged as a popular method for discovering potent compounds due to the inefficiency of lab-based high throughput screening (HTS) (Jin et al., 2020; Kandeel and Al-Nazawi, 2020). VS for rational drug discovery is essentially an approach that involves computationally targeting a specific biomolecule (e.g., DNA, protein, RNA, lipid) of a cell to inhibit its growth and/or activation (Shoichet, 2004; Lionta et al., 2014). Structure-based and ligand-based drug discovery and design are two important subgroups of this type of screening (Lionta et al., 2014; Yu and Mackerell, 2017; Arshadi et al., 2020; Broom et al., 2020). Given our access to computationally and experimentally determined viral protein structures (Senior et al., 2020; Zhang L. et al., 2020), VS provides a rapid and cost-effective strategy for identifying antiviral candidates.

Additionally, conventional vaccine discovery methods have been costly, and it may take many years to develop an appropriate vaccine against a specified pathogen. In the early 1990s, the introduction of a genome-based vaccine design approach dubbed “Reverse Vaccinology” (RV) (Rappuoli, 2000; Bullock et al., 2020), revolutionized the field to a more efficient status, due in part to the fact that bacterial culturing was no longer required for identifying vaccine targets (Bruno et al., 2015; Heinson et al., 2015; Soria-Guerra et al., 2015). Moreover, all of the putative target protein antigens can be identified, rather than identification being limited to those isolated from bacterial cultures (Xiang and He, 2009; Bowman et al., 2011). All of these advantages taken together led scientists to generate RV prediction programs.

Over the past decade, artificial intelligence (AI)-based models have revolutionized drug discovery in general (Zhong et al., 2018; Duan et al., 2019; Lavecchia, 2019). AI has also led to the creation of many RV virtual frameworks, which are generally classified as rule-based filtering models (Naz et al., 2019; Ong et al., 2020a). Machine learning (ML) enables the creation of models that learn and generalize the patterns within the available data and can make inferences from previously unseen data. With the advent of deep learning (DL), the learning procedure can also include automatic feature extraction from raw data (Lecun et al., 2015). Moreover, it has recently been found that deep learning's feature extraction can result in superior performance compared to other computer-aided models (Ma et al., 2015; Chen et al., 2018; Zhavoronkov et al., 2019).

In this review, we provide a survey of AI-based models for COVID-19 drug discovery and vaccine development. Moreover, we identify and evaluate the best candidate targets for future treatment development. We propose that a concerted effort should be made to leverage the knowledge from pre-existing data by using machine learning approaches. To that end, we present a wide-ranging collection of small molecules, peptides, and epitopes for therapy discovery that could also direct AI-based models, screening, or generation, in an intelligent manner.

## Background of Machine Learning Methods for Therapy Discovery

In recent years, machine learning has revolutionized many fields of science and engineering. It has largely transformed our daily lives, from speech and face recognition (Alaghband et al., 2020; Grover and Toghi, 2020; Sun et al., 2020) to customized targeted advertisements (Zhai et al., 2016). The power of automatic abstract feature learning, combined with a massive volume of data, has immensely contributed to the successful application of ML (Lecun et al., 2015). Two of the most impactful areas affected are drug and vaccine discovery (Chen et al., 2018), in which ML has offered compound property prediction (Ma et al., 2015), activity prediction (Zhavoronkov et al., 2019), reaction prediction (Fooshee et al., 2018), and ligand–protein interaction.

On the prediction front, Graph Convolutional Neural Networks (GCNN) have been the favorite tool for drug discovery applications (Duvenaud et al., 2015; Kearnes et al., 2016). These networks are able to handle graphs and extract features via encoding the adjacency information within the features. Successful representation learning from molecules using GCNNs has been demonstrated in drug property prediction (Heskett et al., 2018; Bazgir et al., 2019; Liu et al., 2019), protein interface estimation (Fout et al., 2017), reactivity prediction (Coley et al., 2019), and drug–target interactions (Torng and Altman, 2019; Wang et al., 2020). Sequence-based models such as genomics, proteomics, and transcriptomics have also gained some attention in recent years due to the advancements made in the natural language processing domain. The more recent generation of context-based models are transformers that use attention mechanisms and self-supervision to extract representations from sequences (Vaswani et al., 2017; Devlin et al., 2018). Transformers have demonstrated the capacity to predict drug–target interactions (Shin et al., 2019), model protein sequences (Choromanski et al., 2020), and predict retrosynthetic reactions. These models learn to extract features from sequences on the location, context, and order of the input tokens (Belinkov and Glass, 2018). Recurrent neural networks (RNNs) and long short-term memory (LSTM) networks have successfully demonstrated the ability to perform when trained on molecules or protein sequences to predict secondary structure (Pollastri et al., 2002), quantitative structure–activity relationship (QSAR) modeling (Chakravarti et al., 2019), and function prediction (Liu, 2017).

On the lead generation front, *de novo* design has benefitted the most from the application of deep learning. This subfield has drastically evolved from its traditional usage of ligand-based models and creating molecules from sub-blocks (Acharya et al., 2010). The current approach involves the use of state-of-the-art deep learning models such as Generative Adversarial Networks (GANs) to create data-oriented molecules (Guimaraes et al., 2017). Traditional *de novo* design fails to fully implement this exploration by constraining the generation of molecules with ligand or fragment libraries. More recent approaches utilize deep learning generative models such as variational autoencoders (VAE) (De Cao and Kipf, 2018) in order to create sequences of atoms. This approach lifts the constraints of ligand-based designs and allows the generation of unique molecules with greater diversity (Guimaraes et al., 2017; De Cao and Kipf, 2018; Jin et al., 2018; Liu et al., 2018; Simonovsky and Komodakis, 2018).

Machine learning has also improved the field of vaccine design over the past two decades. VaxiJen was the first implementation of ML in RV approaches and has shown promising results for antigen prediction (Doytchinova and Flower, 2007; Heinson et al., 2017). In addition, the recent development of Vaxign-ML, a web-based RV program leveraging machine learning approaches for bacterial antigen prediction, is a testament to the success of exercising mathematical ML-based in RV (He et al., 2010a; Heinson et al., 2017). In essence, these pipelines consist of feature extraction, feature selection, data augmentation, and cross-validation implemented to predict vaccine candidates against various bacterial and viral pathogens known to cause infectious disease. The use of biological, structural, and physiochemical features is prevalent among the approaches in this domain, as seen in reverse vaccinology and immunoinformatic methods such as IEDB and BlastP, which are feature extractors for AI-based models like RNN in the study of different pathogenic viruses (Flower et al., 2010; He and Zhu, 2015; Abbasi, 2020). More recently, graph-based features have also shown the ability to represent the antibodies instead of an expert-designed feature; Magar et al. showed that graph featurization is followed by mean pooling, and then classification is implemented using shallow and deep models (Magar et al., 2020). Deep Learning approaches have also revolutionized the field of cancer vaccinology through the improved prediction of neoantigens and their HLA binding affinity (Sher et al., 2017; Tran et al., 2019; Wu et al., 2019). Autoencoders of deep learning have shown promising improvement in extracting characteristics of human Leukocyte Antigen (HLA-A), which could be utilized in both transplantations and vaccine discovery (Miyake et al., 2018).

Key aspects of therapy discovery are safety and reliability. The Vaccine Adverse Event Reporting System (VAERS) and Vaccine Safety Databank (VSD) have been among the most popular immunization registries for tracking, recording, and predicting vaccine safety. In prior decades, implementations of computational simulation and mathematical modeling have significantly improved the tradeoff between the assessment of safety and efficacy by using the aforementioned resources (He et al., 2010b; Vaishnav et al., 2015). Zheng et al. implemented Natural language Processing (NLP) for the identification of adverse events related to Tdap vaccines (Zheng et al., 2019).

In drug development cases, the final drug candidate produced in the process of drug discovery needs to be safe for human consumption. This requires an observation of the drug's side effects as well as confirmation that the drug is non-toxic. To accomplish this, the Toxicology in the 21st Century program (Tox-21) has screened ~10,000 compounds from 70 screening assays, creating a database that can be used to facilitate toxicity modeling. Furthermore, the project has also expanded to contain 700 assays with nearly 1,800 molecules in the ToxCast dataset. On the side-effect prevention front, the off-target interactions are predicted and minimized *in silico*. In doing so, potential drug candidates are chosen, with consideration given to their off-target polypharmacological profiles (Zhou H. et al., 2015). In a different approach, AI-based studies were implemented to detect the potential prolongation of QT intervals and cardiotoxicity of a candidate drug, hydroxychloroquine, using ECG data from smartwatches (Li J. et al., 2020)^1^.

In summary, artificial intelligence has been applied to many subfields of drug discovery and vaccine development. This improvement is crucial for the current situation and immediate SARS-COV-2 therapy discovery for several key reasons. Firstly, the automatic feature extraction ability of deep learning can support models with better accuracy and deliver more reliable results. Secondly, the generative ability demonstrated by deep learning models can be utilized to create more druggable molecules and better epitope prediction, lowering the chance of failure in the trial pipeline. Lastly, the novelty of the virus causes the data around its possible therapies to be scarce, which is a suitable scenario for transfer learning and leveraging the learned knowledge from previous tasks (e.g., TranscreenTM) (Salem et al., 2020). Transfer learning has been shown to alleviate this problem through the transferring of learned knowledge and parameters from a secondary task with big data available to the task at hand (Weiss et al., 2016). Therefore, the use of deep learning in therapy discovery for SARS-COV-2 is essential in order to make a timely and accurate response to the virus.

## COVID-19 Molecular Mechanism and Target Selection

Coronaviruses are enveloped viruses with a positive-sense single-stranded RNA genome (Fehr and Perlman, 2015). They are known to infect both humans and other eukaryotes (Andersen et al., 2020; Hoffmann et al., 2020). The novel coronavirus manages to bind to the host receptor with a higher affinity than SARS due to the increased modification of its viral spike, among other structural proteins, resulting in enhanced transmission (Zhou Y. et al., 2020).

**Figure 1:**
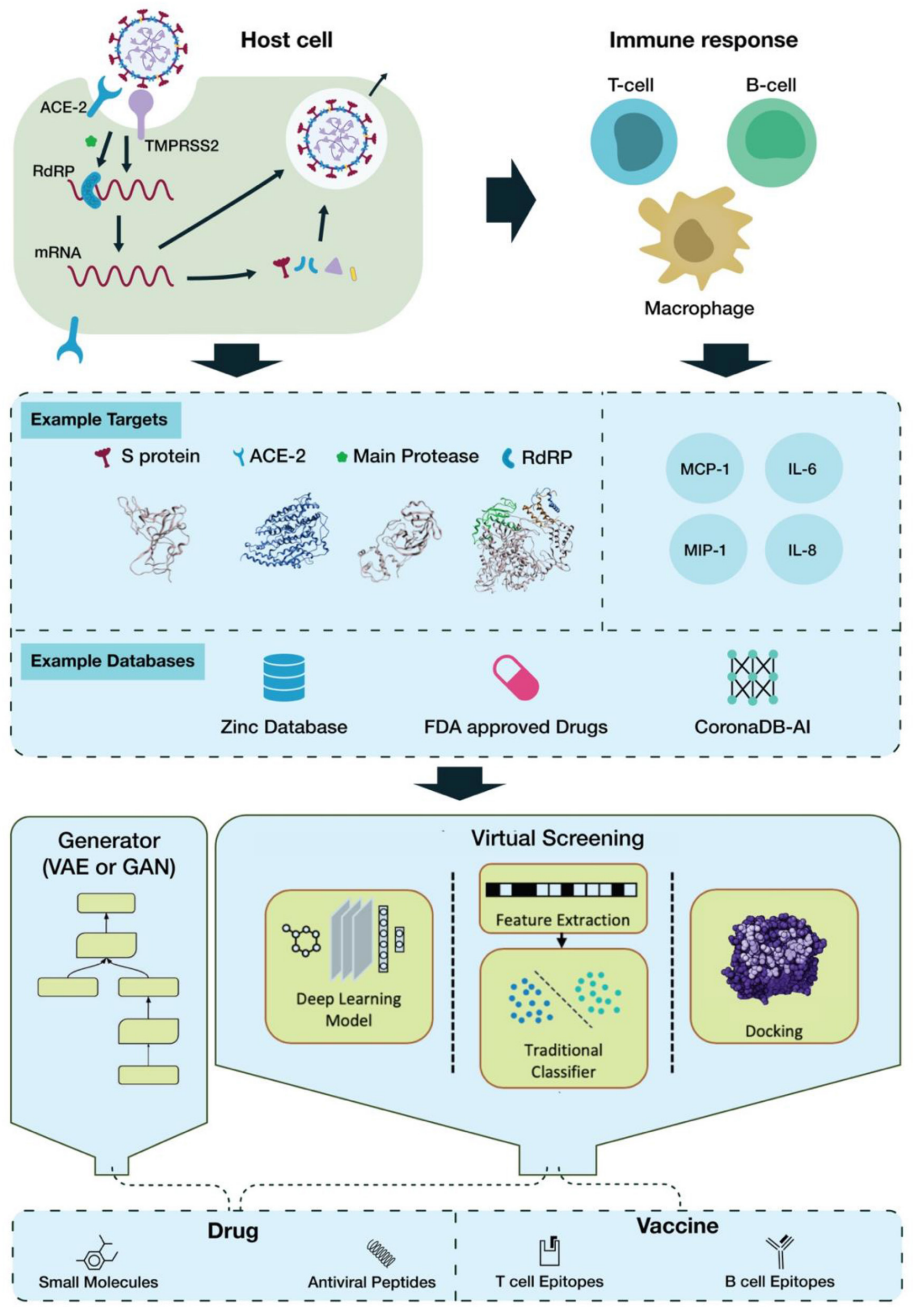
The pipeline of AI-based drug discovery and vaccine development for COVID-19.

SARS-CoV-2 interaction with host cells begins with attachment via the viral spike (S) protein to the host ACE2 receptor (Hoffmann et al., 2020; Zhou P. et al., 2020). ACE2 binding induces the host surface serine protease, TMPRSS2, to prime the S protein via cleavage at its S1/S2 border, facilitating viral fusion with the cell membrane (Hoffmann et al., 2020). Once inside the cell, the viral RNA genome is released into the cytosol, where it is translated by host ribosome machinery, producing two polyproteins: pp1a and pp1ab, which are then cleaved by viral 3CL protease (main protease) and PL protease. This gives rise to several non-structural proteins (nsps) as the foundation of RNA-dependent RNA polymerase (RdRP); this RdRP then transcribes a template strand of the genomic RNA, from which it then transcribes subgenomic mRNA products to be translated. These products encode the structural proteins S, E, M, and N, as well as additional accessory nsps (Figure 1) (Lai and Cavanagh, 1997; Kim D. et al., 2020).

The severity of the host response depends on an innate response to viral recognition, involving the expression of type-1 IFNs and pro-inflammatory cytokines (Pazhouhandeh et al., 2018; Prompetchara et al., 2020). If the antiviral response is delayed or inhibited, viral proliferation can lead to the large-scale recruitment of neutrophils and monocyte-macrophages to the lungs, creating a hyperinflammatory environment (Prompetchara et al., 2020). Overactive release of pro-inflammatory cytokines, i.e., cytokine storm (CS), has been found in COVID-19 patients and can lead to severe complications like acute respiratory distress syndrome (ARDS) (Moore and June, 2020). It has been found that levels of IL-1B, IL-1RA, IL-8, IL-10, IFNγ, IP10, MCP1, and MIP1s are higher in COVID-19 patients than in healthy adults (Huang et al., 2020). IL-6, in particular, has been highly implicated in CRS and COVID-19 severity, and inhibition of IL-6/IL-6R activity may lead to improved patient outcome, increasing its desirability as a target (Figure 1) (Scheller et al., 2014; Tanaka et al., 2016; Zhang C. et al., 2020).

Throughout the process of viral entry, replication, and dissemination, there are several proteins that can serve as suitable targets for therapeutic intervention. The S protein is one of the candidates receiving the most focus, as it is necessary for viral entry into host cells and is highly specific to the virus itself. The host receptor ACE2 is another possible target, but the presence of ACE2 in non-lung tissues such as heart, kidney, and intestine (Hamming et al., 2004) could complicate its inhibition. Another host protein, the TMPRSS2 protease, is essential for viral entry into the cell, making it an additional viable target (Hoffmann et al., 2020).

## COVID-19 Drug Discovery

### Protein-Based

The recent applications of Artificial Intelligence for COVID-19 include the virtual screening of both repurposed drug candidates and new chemical entities. For repurposed drugs, the goal has been to rapidly predict and exploit interconnected biological pathways or the off-target biology of existing medicines that are proven safe and can thus be readily tested in new clinical trials. In one of the early attempts, Gordon et al. paved the way for the repurposing of candidate drugs by experimentally identifying 66 human proteins linked with 26 SARS-CoV-2 proteins (Gordon et al., 2020). In addition to wet-lab approaches, network-based model simulation has been the main computational approach for analyzing the virus–host interactome (Messina et al., 2020). Li et al. identified 30 drugs for repurposing by analyzing the genome sequence of three main viral family members of the coronavirus and then relating them to the human disease-based pathways (Li X. et al., 2020). In a different approach, Zhou et al. offered a combination of network-based methodologies for repurposed drug combination (Zhou Y. et al., 2020).

UK-based BenevolentAI leveraged its AI-derived knowledge graph, which integrates biomedical data from structured and unstructured sources (Richardson et al., 2020). It targeted the inhibition of host protein AAK1 and identified Baricitinib, an approved drug for the treatment of rheumatoid arthritis (Stebbing et al., 2020). Similarly, Beck et al. published an application of their DL-based drug–target interaction model that predicted commercially available antiviral drugs that may target the SARS-COV-2-related protease and helicase (Beck et al., 2020a). Atomwise has also focused on targeting several SARS-CoV-2 protein binding sites that are highly conserved across multiple coronavirus species in an effort to develop new broad-spectrum antivirals. Using its *AtomNet*® deep convolutional neural network technology (Wallach et al., 2020), Atomwise is screening millions of virtual compounds against these diverse targets alongside 15 different partnerships with academic researchers that will test the predicted compounds in their *in vitro* assays^2^.

There have been several other applications of multi-task deep learning models for identifying existing drugs that can target the main viral proteins, especially the main protease (3CL^pro^) and spike protein (Hu et al., 2020; Kadioglu et al., 2020; Kim J. et al., 2020; Redka et al., 2020). One impressive example is Cyclica's creation and mining of PolypharmDB, a platform of known drugs and their predicted binding to human protein targets that uncovered off-target applications of 30 existing drugs against the viral protein 3CL^pro^ and the ACE2 binding site as two examples (Redka et al., 2020). At least two other applications of DL-based virtual screening for the SARS-CoV-2 main protease have been published and include the open sharing of newly predicted chemical structures (Bung et al., 2020; Zhang H. et al., 2020).

ML-aided molecular docking has been one of the most prevalent approaches for virtual screening. This process normally requires the following: (1) Dataset of Druglike or Approved Molecules, (2) Crystal Structure or Homology Model of the target, (3) Molecular Docking Program, and (4) Compute Resources (Ewing et al., 2001; Pagadala et al., 2017). Through docking, many molecules have been reported to fit the binding site of various SARS-CoV-2 proteins essential for viral replication and infection. 3CL^pro^, Spike Protein, RdRP, and PL^pro^ are among those screened, as well as the host ACE2 receptor and TMPRSS2 protease (Chen et al., 2020; Choudhary et al., 2020; Kong R. et al., 2020; Smith and Smith, 2020; Wu et al., 2020). As an example, Ton et al. identified at least 1000 protease inhibitors by creating and utilizing the Deep Docking (DD) network technology approach. However, as they used the QSAR for training their model, no novel docking score was provided (Ton et al., 2020).

It is clear that 3CL^pro^ is the most popular target for virtual screening (Figure 1). The main reason for this is its pivotal role in viral replication and transcription and its well-defined structural information. Viral protease inhibitors have been extensively studied as treatments for other viruses. In addition, deep learning-aided approaches have been the main focus of research, as their automatic feature extraction accelerates discovery. The datasets cited often rely on the ZINC database (Wu et al., 2020), while other screened datasets include the FDA-approved LOPAC library (Choudhary et al., 2020), SWEETLEAD library (Smith and Smith, 2020), or all purchasable drugs (Drugs-lib) (Chen et al., 2020). Moreover this review sampled a variety of publications witch used different computational resources. It can be carried out on a small scale on a MacOS Mojave Workstation with an 8 core Zeon E5 processor or on a large scale as with the world's strongest supercomputer, SUMMIT, for enhanced parallelization (Choudhary et al., 2020; Smith and Smith, 2020).

### RNA-Based

Conserved structured elements have already been shown to play critical functional roles in the life cycles of Coronaviruses (Yang and Leibowitz, 2015). Through direct interactions with host RNA-binding proteins and helicases, structural elements add a layer of complexity to the regulatory information that is encoded in the viral RNA. Targeted disruption of the regulatory functions of these structural elements provides a largely unexplored strategy that can limit viral loads with minimal impact on the biology of normal cells (Park et al., 2011). While this idea would have been farfetched a mere 5 years ago, advances in AI-driven computational modeling and high-throughput experimental RNA shape analyses have all but overcome the critical barriers (Alipanahi et al., 2015).

Highly conserved RNA structural elements have been identified in a number of viral families, many of which have been functionally validated (Jaafar and Kieft, 2019). Some of these stem loops in SARS-CoV-2′s 5′UTRs structural elements are conserved across beta coronaviruses and are known to impact viral replication (Yang and Leibowitz, 2015). There are many functional RNA structural elements that fall within the coding sequence and the 3′UTR as well (Plant and Dinman, 2008; Stammler et al., 2011). Rangan et al. identified 106 structurally conserved regions that would be suitable biotargets for unexplored antiviral agents (Rangan et al., 2020). Moreover, they predicted at least 59 unstructured regions that are conserved within SARS-CoV-2. Park et al. identified an RNA Pseudoknot-Binding molecule against SARS-CoV-1 in target-based virtual screening (Park et al., 2011; Nakagawa et al., 2016).

Studying the changes in RNA information also allows for the identification of new and evolved targets. In a different approach, Wu et al. showed that a recently FDA-approved drug named Remdesivir could bind to the RNA-binding channel of the novel coronavirus. They discovered other candidate drugs via analyzing the proteins critical to RNA processing and pathways (Wu et al., 2020). It seems that viral genome, RdRP, and processed mRNA would make promising targets for drug repurposing.

### Generative Approaches

Molecule generation has been one of the fields of drug discovery that have been most revolutionized by the implementation of artificial intelligence over the last decade. As mentioned, VAE is a generator model for enhancing the diversity of generated data. Autoencoders instruct molecules into a vector that captures properties such as bond order, element, and functional group (Bjerrum and Sattarov, 2018). Chenthamarakshan et al., together with IBM Research, demonstrated a VAE that captures molecules in a latent space. Once captured, variations are made on the original molecule vectors based on desired properties. These can then be decoded back into novel molecules (Chenthamarakshan et al., 2020). To optimize the structures, QED, Synthetic Accessibility, and LogP regressors were used to improve the latent space variations.

In a different approach, Tang et al. overcame many of the issues with traditional generative models by developing a novel advanced deep Q-learning network with fragment-based drug design (ADQN-FBDD). This allowed for the enhanced exploration of space by assembling SARS-CoV-2 molecules one fragment at a time rather than relying on latent space adjustments. After making connections and rewarding molecules with the most druglike connections, a pharmacophore and descriptor filter was used to refine the set. They demonstrated a robust method for designing novel, high-binding compounds refined to the structure of SARS-CoV-2 3CL^Pro^ (Tang et al., 2020). To design a drug-generative network, the following is necessary: (1) collection of Druglike Molecules, (2) a representation of these molecules *in silico* (i.e., Fingerprints, Tokenizers), (3) a method of altering molecules to increase diversity, and (4) screening and modification of the altered molecules. Pursuing GAN-related models, Insilico Medicine used three of its previously validated generative chemistry approaches to target the main protease, namely, crystal-derived pocked-based generation, homology modeling-based generation, and ligand-based generation (Zhavoronkov et al., 2020). Similar to target-based virtual screening, the main protease has been the main object of interest for scientists for *de novo* drug discovery.

## COVID-19 Vaccine Discovery

Identification of the best possible targets for the development of a vaccine is crucial in order to counteract a virus's high infection rate (Choudhary et al., 2020). A host immune system fights virus-infected cells either through the production of antibodies by B cells or through the direct attack of T cells (Amanat and Krammer, 2020). The HLA gene encodes MCH-I and MCH-II proteins, which present epitopes as antigenic determinants. These proteins assist B-cell and T-cell antibodies in their ability to bind and attack invaders (Dangi et al., 2018; Gupta et al., 2020; Smith and Smith, 2020). Machine learning approaches, including Random Forest (RF), Support Vector Machine (SVM), and Recursive Feature Selection (RFE), have been basic tools for identifying antigens from protein sequences (Bowick et al., 2010; Rahman et al., 2019). However, due to their low sensitivity in the prediction of locally clustered interactions in some cases, Deep Convolutional Neural Networks (DCNN) have been a more valid alternative for the binding prediction of MHC and peptides (Han and Kim, 2017).

Since the outbreak of this first coronavirus, different AI-based approaches have been used to predict potential epitopes so as to design vaccines (Park et al., 2011; Yang and Leibowitz, 2015; Ton et al., 2020). Fast and Chen used MARIA (Chen et al., 2019) and NetMHCPan4 (Jurtz et al., 2017), two supervised neural network-driven tools, to discover potential T-cell epitopes for SARS-CoV-2 close to the 2019-nCoV spike receptor-binding domain (RBD) (Fast and Chen, 2020). The Long Short-Term Memory (LSTM) network has also shown some promising results. Abbasi et al. used this type of RNN to predict epitopes for Spike (Abbasi, 2020). Using a similar tactic, Crossman et al. employed deep-learning RNN and provided simulated sequences of Spike to identify possible targets for vaccine design (Crossman, 2020). RNN provided the sequences for a protein of interest with high sequence identity to the BLAST match.

Using a separate method, Feng et al. leveraged the iNeo tool to design a vaccine containing both B-cell and T-cell epitopes. This multi-peptide vaccine could provide a new strategy against SARS-CoV-2. Additionally, they discovered 17 vaccine peptides involving both immune cells (Nakagawa et al., 2016; Rangan et al., 2020). Ong et al. used Vaxign-RV to prioritize non-structural proteins as vaccine candidates for SARS-CoV-2 (Ong et al., 2020b). Nsp3, the largest non-structural protein of the coronavirus family, was identified as the most promising potential target for vaccine development after Spike (Ong et al., 2020b). Malone et al. also studied the entire SARS-CoV-2 proteome beyond Spike and provided a comprehensive vaccine design blueprint for SARS-CoV-2 using *NEC Immune* Profiler, IEDB, and BepiPred tools to create an epitope map for different HLA alleles (Malone et al., 2020).

Natural language processing models, specifically language modeling techniques, have also made an impact in the domain of COVID-19 vaccine discovery. Pre-trained transformers were used to predict protein interaction (Nambiar et al., 2020) and model molecular reactions in carbohydrate chemistry (Pesciullesi et al., 2020), which can be utilized in the process of vaccine development. Chen et al. discussed the use-case of an LSTM-based seq-2-seq model for predicting the secondary structure of certain SARS-COV-2 proteins (Karpov et al., 2019)^3^. Also, Beck et al. used transformers to repurpose commercially available drugs by predicting their interactions with viral proteins of SARS-COV-2 (Beck et al., 2020b).

Taking this work together, it is clear that spike protein has been the most popular candidate for virtual vaccine discovery (Oany et al., 2014). As the spike protein of SARS-COV-2 is crucial for viral entry, specific neutralizing antibodies against the receptor-binding domain of Spike can interrupt the attachment and fusion of viral proteins (Wan et al., 2019). This method could provide simulated sequences that can serve as a guide for further vaccine discovery against COVID-19 and possibly new zoonosis that may arise in the future.

## Data Collection

Data-driven solutions rely on patterns embedded in the data in order to extract mathematical models. That being said, a data collection campaign will face a plethora of challenges in the case of any recently emerged virus, primarily due to the existence of bias and imbalance in the limited data available. Therefore, even the most sophisticated of modeling approaches will be ineffective when trained on such datasets. In order to overcome this issue, we compiled a multifaceted and comprehensive investigation of the existing literature, datasets, and online resources to provide potential small molecules, peptides, and epitopes. Such elements can be beneficial in the process of discovering or designing novel drugs to treat COVID-19 when used with both conventional and data-driven AI-based approaches.

We choose to focus on both potential antiviral agents and host biotarget inhibitors. The provided data entitled CoronaDB-AI in Table 1 includes the small molecules and peptides proposed by both *in-silico* and *in-vitro* approaches. In addition to candidate scaffolds against the coronavirus's structural proteins, the potential inhibition of other respiratory tract viruses is taken into consideration to increase the therapeutic potential. Antimicrobial peptides have been validated as potent antivirals that disrupt either the viral membrane or an additional molecular mechanism of the virus (Akaji et al., 2011; Han and Kraí, 2020; Xia et al., 2020). As described before, the cytokine storm and an elevated immune response of the host plays a vital role in disease complication, so candidate immunosuppressants were also added as host-targeted agents. In addition to the potency of a candidate drug, it is crucial that the drug have high selectivity and low toxicity. Therefore, we also gathered a complete toxicity dataset from distinct databases, including ToxCast and Tox21. Finally, we gathered a comprehensive epitope-based dataset that could also guide deep learning-based models for improved vaccine development and epitope generation.

**Table 1:** CoronaDB-AI is a collection of small molecules, peptides, and epitopes for the purpose of COVID-19 therapy discovery.

**Data provided**	**Discovery**	**Type**	**Mechanism of action**	**References**
**ANTIVIRAL DATA**				
**Total of 59,107**		**Small molecules and peptides**		
50,000	*In-silico*	Small molecule	Antiviral	1
3,000	*In-silico*	Small molecule	Anti SARS2 protein	Chenthamarakshan et al., 2020
1,000	*In-silico*	Small molecule	Anti-protease	Ton et al., 2020
406	*In-vitro*	Small molecule	Inhibiting autophagy	2
802	*In-vitro*	Small molecule	Activating autophagy	2
393	*In-vitro*	Small molecule	Biotargets of coronaviruses	3
110	*In-vitro*	Peptide and small molecule	Coronavirus and respiratory disease	Pillaiyar et al., 2020
1,000	*In-silico*	Small molecule	3C protease inhibitor	Zhavoronkov et al., 2020
11	*In-silico*	Small molecule	Main protease inhibitor	Fischer et al., 2020
20	*In-vitro*	Antimicrobial peptide	Anti-SARS/MERS	Mustafa et al., 2018
7	*In-silico*	Antimicrobial peptide	Anti-MERS	Mustafa et al., 2019
277	*In-vitro*	Antimicrobial peptide	Antiviral	Wang et al., 2015
4	*In-silico*	Antimicrobial peptide	Anti-spike of sars-Cov-2	Han and Kraí, 2020
379	*In-vitro*	Small molecule	Anti-respiratory syncytial virus	Plant et al., 2015
13	*In-vitro*	Small molecule	Anti-recurrent respiratory papillomatosis by HPV-6	Alkhilaiwi et al., 2019
1,280	*In-vitro*	Small molecule	Anti-respiratory syncytial virus	Rasmussen et al., 2011
16	*In-silico*	Small molecules	Anti-SARS-COV-2	Zhou Y. et al., 2020
77	*In-silico*	Small molecules	Anti-S Protein of SARS-COV-2	Smith and Smith, 2020
10	*In-silico*	Small molecules	Anti-SARS-COV2	Hu et al., 2020
25	*In-silico*	Small molecules	Anti SARS2 Proteins	Kim J. et al., 2020
10	*In-silico*	Small molecules	ACE2 and Spike inhibitors	Choudhary et al., 2020
78	*In-silico*	Small molecules	All SARS2 proteins	Wu et al., 2020
47	*In-silico*	Small molecules	3cl protease and M pro	Tang et al., 2020
16	*In-silico*	Small molecules	3cl protease inhibitor	Chen et al., 2020
36	*In-vitro*	Small molecules	Anti- Coronavirus-OC43	Shen et al., 2019
90	*In-vitro*	Small molecules	Anti- SARS-COV-2	Touret et al., 2020
**ANTI-HOST PROTEINS**				
**Total of 677**		**Small molecules and peptides**		
6	*In-vitro*	Small molecules	Anti-IL-1β and TNFα	Laufer et al., 2002
182	*In-vitro*	Peptides	Cytokine Signaling Inhibitors	4
269	*In-silico*	Small molecules	Anti-IL-6	Shukla et al., 2019
121	*In-vitro*	Small molecules	Severe acute respiratory	5
69	*In-silico*	Small molecules	Anti-protein-protein interaction of virus-host	Gordon et al., 2020
30	*In-silico*	Small molecules	Anti-host & virus interaction	Redka et al., 2020
**TOXICITY DATA**				
**Total of 25,333**		**Small molecules**		
11,800	*In-vitro*	Small molecules	Tox21 and ToxCast	Toxicology, EPA's National Center for Computational, 2018
13,533	*In-vitro*	Small molecules	Toxic for HepG2 Cell Line	Gamo et al., 2010
**VACCINE DATA**				
**Total of 517**		**Epitopes and vaccines**		
162	*In-silico*	Epitopes	Anti-SARS-COV-2	Ahmed et al., 2020
174	*In-silico*	Epitope	Anti-SARS-COV-2	Prachar et al., 2020
2	*In-silico*	Epitope	Anti-SARS-COV-2	Fast and Chen, 2020
30	*In-silico*	Vaccine candidate	Anti-SARS-COV-2	Feng et al., 2020
7	*In-silico*	Epitope	Anti-SARS-COV-2	Lon et al., 2020
12	*In-silico*	Epitope	Anti-SARS-COV-2	Tilocca et al., 2020
59	*In-silico*	Epitope	Anti-SARS-COV-2	Sarkar et al., 2020
71	*In-silico*	Epitope	Anti-SARS-COV-2	Bhattacharya et al., 2020

*^1^Download CAS COVID-19 Antiviral Candidate Compounds Dataset | CAS. Available online at: https://www.cas.org/covid-19-antiviral-compounds-dataset (accessed April 27, 2020)*.

*^2^Novel Coronavirus Information Center. Available online at: https://www.elsevier.com/connect/coronavirus-information-center (accessed April 27, 2020)*.

*^3^https://www.elsevier.com/__data/assets/pdf_file/0004/978745/Copy-of-RMC-substances-coronovirus-targets-pX6.pdf (accessed April 27, 2020)*.

*^4^Cytokines Inhibitor library|Targetmol|96-well. Available online at: https://www.targetmol.com/compound-library/Cytokines-inhibitors-Library (accessed April 27, 2020)*.

*^5^https://www.elsevier.com/__data/assets/pdf_file/0007/977173/ResNet-Data_Coronavirus.pdf (accessed April 27, 2020)*.

## Discussion

SARS-COV-2 rapidly transformed into a global challenge, costing thousands of lives, overwhelming healthcare systems, and threatening the economy all around the world. As we demonstrated above, it can be extremely challenging to experimentally perform a comprehensive potency evaluation of all drug and vaccine candidates in a timely fashion. We believe that leveraging computational models capable of filtering and generating reliable therapies can significantly speed up these discovery efforts. Employing artificial neural networks and supervised learning methods has proven to be a vital game-changer when used for the purpose of virtual filtering and *de novo* design. However, in order to achieve the desired performance in such intelligent methods, one requires the knowledge to recognize the most relevant biotargets in addition to a large-scale training dataset. This fact motivated us to perform a survey of biotargets that have been employed in the virtual drug and vaccine discovery literature. We observed that the viral spike protein and the main protease have been the most prevalent choices for vaccine development and drug discovery, respectively, due to their importance. Furthermore, we gathered a list of datasets titled “CoronaDB-AI” that can be used for our particular application. Having access to these key elements removes the burden of collecting training data and the required knowledge for both computer scientists and bioinformaticians and consequently enhances research outcomes.

## Author Contributions

AK organized and wrote most of article and gathered all the data. JW contributed to the molecular part. MS contributed to the background for AI-based methods. EC, ED-C, and BK from A2A and SC-T from Atomwise contributed to the COVID19 drug discovery. NG and JC contributed to the vaccine discovery. HG contributed to the RNA-based and molecular sections. JY provided guidance in the opportunities of deep learning in a multidiscipline collaboration. All authors contributed to the article and approved the submitted version.

## Conflict of Interest

EC, ED-C, and BK were employed by the company A2A Pharmaceuticals. SC-T was employed by the company Atomwise Inc. The remaining authors declare that the research was conducted in the absence of any commercial or financial relationships that could be construed as a potential conflict of interest.

## Footnotes

^1^AI study launched to monitor cardiac safety of COVID-19 patients receiving hydroxychloroquine. Available online at: https://cardiacrhythmnews.com/ai-study-launched-to-monitor-cardiac-safety-of-covid-19-patients-receiving-hydroxychloroquine/ (accessed July 04, 2020).

^2^Atomwise Partners with Global Research Teams to Pursue Broad-Spectrum Treatments Against COVID-19 and Future Coronavirus Outbreaks | Business Wire. Available online at: https://www.businesswire.com/news/home/20200521005238/en/Atomwise-Partners-Global-Research-Teams-Pursue-Broad-Spectrum (accessed June 28, 2020).

^3^OSF Preprints. ZeroFold-Understanding Mutations of SARS-CoV-2 Spike Protein base on Secondary Structure Event Extracting for guiding Vaccine development. Available online at: https://osf.io/3vkuw/ (accessed Jul. 01, 2020).
